# Perfluorooctanoic Acid Affects Thyroid Follicles in Common Carp (*Cyprinus carpio*)

**DOI:** 10.3390/ijerph19159049

**Published:** 2022-07-25

**Authors:** Maurizio Manera, Giuseppe Castaldelli, Luisa Giari

**Affiliations:** 1 Faculty of Biosciences, Food and Environmental Technologies, University of Teramo, St. R. Balzarini 1, 64100 Teramo, Italy; 2 Department of Environmental and Prevention Sciences, University of Ferrara, St. Borsari 46, 44121 Ferrara, Italy; giuseppe.castaldelli@unife.it (G.C.); luisa.giari@unife.it (L.G.)

**Keywords:** per- and polyfluoroalkyl substances, persistent organic pollutants, endocrine disruptors, biomarker, fish model

## Abstract

Carp kidney is comprised of nephrons, hemopoietic tissue, and also hormonally-active thyroid follicles. Given this anatomical trait, it has been used to assess the thyroid disrupting potential of perfluorooctanoic acid (PFOA), a widespread and feared per- poly-fluoroalkyl substance and a persistent organic pollutant capable of interfering with the endocrine system in animals and humans. The occurrence and morphology of thyroid follicles in kidneys of carp experimentally exposed to 200 ng L^−1^ or 2 mg L^−1^ waterborne PFOA for 56 days were studied. The abundance of thyroid follicles was significantly higher and vesiculation increased in exposed fish as compared to controls. The number of vesiculated follicles/total number of follicles was positively correlated with PFOA blood concentration in fish exposed to the highest dose (2 mg L^−1^). The structure and ultrastructure of thyroid follicles were affected by PFOA also at the lower, environmentally relevant, concentration (200 ng L^−1^). Increased cellular projections, enhanced colloid endocytosis, rough endoplasmic reticulum enlargement and fragmentation and cytoplasm vacuolation were the main features displayed by PFOA-exposed carp. These results show that PFOA affects the occurrence and status of follicles and suggest the utility of fish kidney as a multipurpose biomarker organ in environmental pathology research, according to the One Health approach.

## 1. Introduction

Perfluorooctanoic acid (PFOA) is among the most studied and representative per- and polyfluoroalkyl substances (PFAS), a wide and heterogeneous class of man-made chemicals defined by the Organization for Economic Cooperation and Development (OECD) as “fluorinated substances that contain at least one fully fluorinated methyl or methylene carbon atom” [1]. PFOA (molecular formula C_8_HF_15_O_2_) has a hydrophobic 7-carbon chain, in which carbon atoms are substituted by fluorine atoms, and a hydrophilic carboxylic group, and as a consequence shows the typical PFAS feature of amphiphilicity [2].

PFAS have been produced since 1950’ and used in more than 200 application areas from fire-suppressing foams to food-packaging and textile treatments, being incorporated in numerous products [3]. PFAS are released in the environment during all their life cycle (i.e., industrial production, commercial use, consume and disposal) and, thanks also to their extreme stability and mobility, are now ubiquitous contaminants occurring in abiotic and biotic matrices [4]. The increasing scientific and public concern about environmental persistence, high exposure risk and adverse effects on wildlife and human populations led in the last years to the regulatory limitation of some older long-chain PFAS (such as PFOA) and the development of next-generation short-chain PFAS that have been poorly characterized to this point [4].

Among the harmful health outcomes of PFOA and other PFAS, they also possess an endocrine toxicity potential, and a recent review summarized the available literature on this topic concluding that further research efforts are necessary to clarify the mechanisms involved and the concentrations/doses at which the endocrine-disrupting action is active [2,5]. Both in vivo and in vitro studies on different test organisms and cells have documented the ability of PFAS to alter steroidogenesis and the binding to the estrogen receptor, androgen receptor and thyroid hormone receptor [2,6]. PFOA interference with the expression or activity of aromatase, a key enzyme for estrogen synthesis from androgens, and PFOA estrogen-like activity have been reported in fish [7,8,9,10].

The thyroid plays a pivotal role in regulation of metabolism, development, growth and reproduction in all vertebrates and its disturbance could cause disorders with negative outcomes at the population level [6]. Changes in thyroid structure/function and in thyroid-related genes resulting in altered hormone levels have been observed in aquatic organisms as organ and molecular responses to PFAS [6]. Lee et al. showed the degeneration and atrophy of thyroid follicular cells in a multi-generational PFOA exposure of *Oryzias latipes*, suggesting a causal link between thyroid abnormality and PFOA [11]. In experimentally exposed zebrafish, PFOA up-regulated the thyroid hormone receptor interactor 13 gene in females and down-regulated the iodothyronine type 2 gene in both males and females [12]. Animals are widely used as experimental models for human diseases and as indicator species for assessing the exposure of both humans and animals to environmental pollutants, according to the One Health approach [13,14]. Teleost fish and other vertebrates share similar thyroid structure, based on a follicular arrangement, and function with the primary hormone secreted being T4 and the histological exam of follicles morphology can provide several indices of thyroid activity [15]. In mammals the thyroid is a compact and encapsulated gland, while in teleost fish the thyroid consists of non-encapsulated follicles scattered in the subpharyngeal region and, in common carp (*Cyprinus carpio*) and other cyprinids, also in other tissues and organs [16]. Carp kidney is composed of nephrons, hematopoietic tissue and also hormonally-active thyroid follicles [17]. For this reason, the renal tissue of carp has been used in the present study to assess the thyroid disrupting potential of PFOA, and is proposed as an organ model to explore, besides the nephrotoxicity and immunotoxicity [18], the endocrine toxicity of PFAS. To our knowledge, this work is one of few documenting the morphological alteration of thyroid in fish exposed to PFOA and the first to take into account the ultrastructural pathology given the importance of electron microscopy to assess toxicity [19]. It should be stressed that toxicologic pathology still relies primarily on lesions to arrive at a diagnosis, where a lesion is the morphological evidence (at the ultrastructural, microscopic or macroscopic levels) of a disrupted function [20]. The main aim is to expand the knowledge of the effects of PFAS on the thyroid, at environmentally relevant concentrations, helping us to understand the risk posed by PFOA as an endocrine disruptor according to the One Health approach.

## 2. Materials and Methods

Histological samples examined for this study were obtained from a previous one [21]. The experimental design of the exposure test, fish biometry, and analytical chemistry used to quantify PFOA concentrations in fish tissues/organs and the light and ultrastructural microscopy methods were reported elsewhere [18,21] and are summarised in brief below.

### 2.1. Experimental Design

Thirty-one two-year-old common carp (total length, mean ± standard deviation: 19.32 ± 2.49 cm; body mass, mean ± standard deviation: 104.84 ± 27.80 g) were divided into three groups: an unexposed control group (Ctr; *n* = 10), and groups exposed to 200 ng L^−1^ PFOA (Low concentration; *n* = 10), and to 2 mg L^−1^ (High concentration; *n* = 11) in a flow through open system (500 mL of water min^−1^). The dose of 200 ng L^−1^ was chosen as an environmentally relevant concentration based on PFOA reports in surface water [22], while the dose of 2 mg L^−1^ was chosen to induce a histological response according to previous research in cyprinid fish [7]. Fish were euthanized by anesthesia with tricaine methanesulfonate (MS-222) followed by spinal cord severing after a sub-chronic exposure of 56 days.

### 2.2. Kidney Tissue Processing for Light and Transmission Electron Microscopy

Kidney samples were fixed in 10% neutral buffered formalin, dehydrated, clarified, paraffin embedded, sectioned at 5 µm, stained with hematoxylin and eosin (H&E) and the Alcian Blue-PAS method, thereafter observed and photographed at light microscopy using a Nikon Eclipse 80i microscope (Nikon, Tokyo, Japan).

For ultrastructural analysis, kidney samples were fixed in 2.5% glutaraldehyde buffered with sodium cacodylate (pH 7.3) at 4 °C for 3 h, post-fixed in 1% osmium tetroxide for 2 h, dehydrated in a graded series of acetone, and embedded in epoxy resin (Durcupan™ ACM, Fluka, Sigma-Aldrich, St. Louis, MO, USA). Ultrathin sections (90 nm) were contrasted with uranyl acetate and lead citrate and examined under a Hitachi H-800 transmission electron microscope (Hitachi Ltd., Tokyo, Japan).

### 2.3. Chemical Analysis

After extraction using an ion-pairing procedure, PFOA was measured in tissues by high-performance liquid chromatography with electrospray ionization tandem mass spectrometry with a level of detection (LOD), determined as three times the signal-to-noise (S/N) ratio, of 0.4 ng g^−1^ wet weight. Standards for the five-point calibration curve were prepared by progressive dilution with methanol from a neat standard and concentrations were evaluated in comparison to this unextracted standard curve and were not corrected for the recoveries or for the purity of the standard (more than 98%). Blanks were analysed with each set of five tissue samples as a check for possible laboratory contamination and interferences; recoveries, assessed using spiked matrix with a concentration of 5 ng g^−1^, were over 89%. The reproducibility was also determined and expressed as the relative standard deviation (RSD) for each concentration level (PFOA 1.9%) [23,24].

### 2.4. Follicle Biometry

Ten sections from 10 carps of the control group and 10 sections from 10 carps of each of the two exposed groups (10 control, 10 low concentration, 10 high concentration; total 30 fish) were screened for thyroid follicles via light microscopy at 400× magnification using image analysis software (Nis Elements AR 3.0; Nikon, Tokyo, Japan). Each tissue section was examined without knowledge of the exposure group and screened in a zig-zag trajectory, avoiding duplicate follicle counts, empty spaces, and non-homogeneous tissue in order to ensure replicability. Moreover, each follicle was classified according to the presence or absence of vesiculations in thyroid epithelial cell and/or colloid in two groups: vesiculated or not vesiculated. As a cut-off value, follicles with a total number of vesiculations less than 1/8 (12.5%) of their entire outline were considered as not vesiculated.

### 2.5. Statistical Analysis

Numerical data were assessed for normality and variance homogeneity by means of a Shapiro-Wilk test and Levene’s test, respectively, and then tested for differences, according to the exposure group and to the follicle types with the proper statistic test. One-way Anova and paired *t*-tests were used as parametric tests, where the normality assumption was met. In particular, a one-way Anova was used to test for differences in absolute numerical values of thyroid follicle types (not vesiculated, vesiculated and total) among the three exposure groups, whereas a paired *t*-test was used to test for differences in relative numerical values (not vesiculated/total, vesiculated/total) within the same exposure group. Accordingly, a Kruskal-Wallis test and Wilcoxon test were used as corresponding non parametric tests, where the normality assumption was not met. Moreover, correlation was tested between the values of renal tissue or blood PFOA concentration and the absolute (not vesiculated, vesiculated and total) and relative numerical values (not vesiculated/total, vesiculated/total) of thyroid follicle type, by means of Pearson’s correlation and Spearman’s correlation, as parametric and non-parametric test, respectively. JAMOVI (JAMOVI 2.3.2, JAMOVI project) and SPSS 14.0.1 (SPSS Inc., Chicago, IL, USA) were used as statistical packages for analysis.

## 3. Results

### 3.1. Follicle Histology

**Figure 1:**
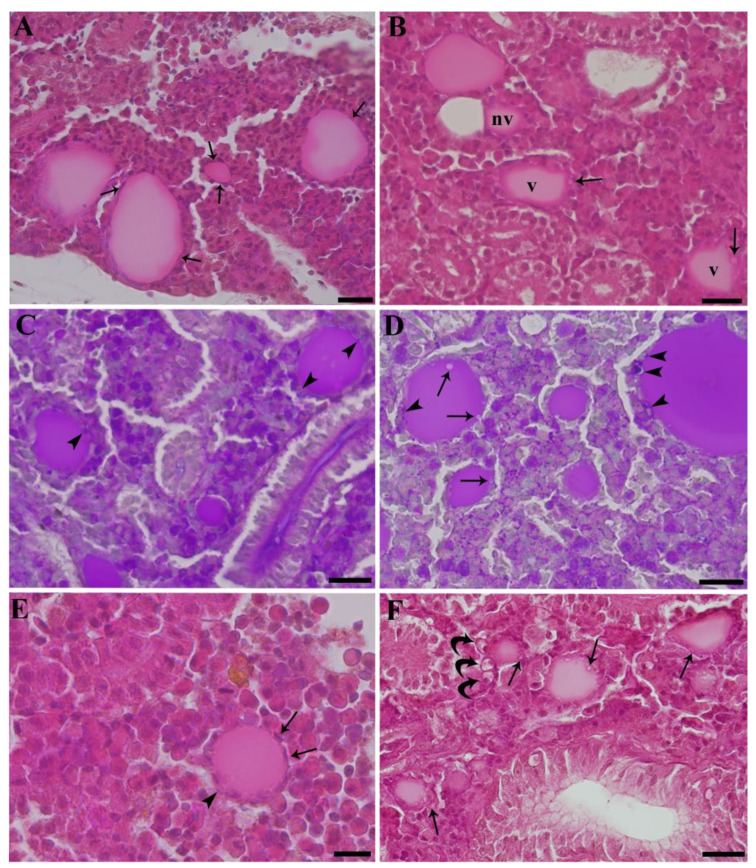
Paraffin-embedded tissue sections of carp kidney. H&E (**A**,**B**,**E**,**F**) and Alcian Blue-PAS (**C**,**D**). (**A**) Unexposed carp. Not vesiculated thyroid follicles. Flattened pavement cells are appreciable (arrows). Scale bar = 22 μm. (**B**) Carp exposed to 2 mg L^−1^ PFOA. Vesiculated (v) and not vesiculated thyroid follicles (nv) are visible in the same microscopic field. The former are lined by cuboidal cells displaying cytoplasm vesiculations (arrows). Scale bar = 22 μm. (**C**) Unexposed carp. Not vesiculated thyroid follicles are lined by a flattened epithelium and show minute PAS positive inclusions (arrowheads), suggestive of endocytized colloid. Scale bar = 22 μm. (**D**) Carp exposed to 2 mg L^−1^ PFOA. More numerous and bigger (in comparison with the unexposed carp in Figure 1C) PAS positive colloid containing phagosomes are appreciable (arrowheads). Vesiculations are also visible (arrows). Scale bar = 22 μm. (**E**) Unexposed carp. A single thyroid follicle is present, displaying part of the epithelium lining flattened, without vesiculations (arrows), and other parts cuboidal, with small vesiculations (arrowhead). Scale bar = 11 μm. (**F**) Carp exposed to 200 ng L^−1^ PFOA. Many small newly formed thyroid follicles are visible. Vesiculations inside the cells are evident (arrows). Three rodlet cells are present close to a vesiculated follicle (curved arrows). Scale bar = 22 μm.

Two main types of thyroid follicle were recognized. In the first type, here referred to as not vesiculated, colloid appeared to be homogeneous and was lined by flattened pavement cells (Figure 1A). In the second one, here referred to as vesiculated, the colloid presented peripheral focal vesiculation and was lined by cuboidal cells displaying cytoplasm vesiculations (Figure 1B). Such cytoplasm vesiculations showed PAS positive content, the same as colloid, suggesting they were phagosomes containing the latter (Figure 1C,D). Follicles displaying intermediate features were also present, with parts of the lining epithelium flattened without vesiculations in colloid and/or in cells, and other parts cuboidal, with evident vesiculations inside the cells and/or in colloid (Figure 1E). Perfluorooctanoic acid exposure affected both the number and the morphology of thyroid follicles. In particular, a higher number of follicles and an enhanced occurrence of vesiculations were observed in PFOA-exposed fish (Figure 1D,F). Notably, in the low concentration group, small newly formed follicles (Figure 1F) were more frequent along with a higher individual variation in vesiculated follicles compared to the other experimental groups.

### 3.2. Follicle Biometry

**Figure 2:**
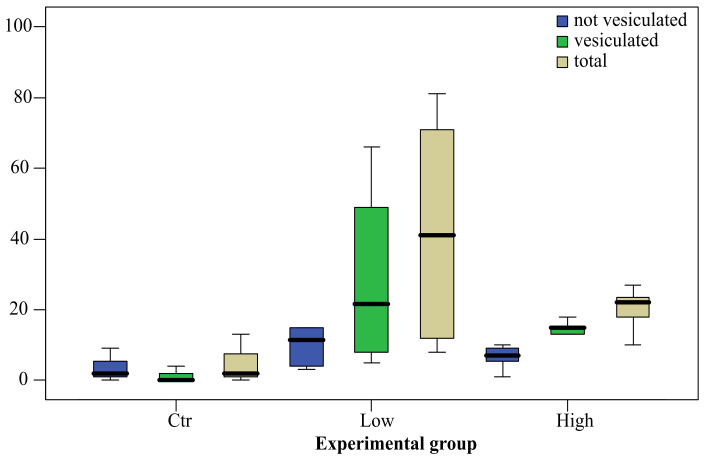
Box plot of the absolute follicles number (in ordinate) according to morphology (not vesiculated, vesiculated and total) and the PFOA exposure group (Ctr, unexposed; Low concentration, 200 ng L^−1^ PFOA; High concentration, 2 mg L^−1^ PFOA). The bold line in the box represents the median and divides the box into a lower and upper quartile (25 percentile). The whiskers represent the upper and lower outer quartiles and, as a consequence, the upper and lower 50 percentile starting from the median.

**Figure 3:**
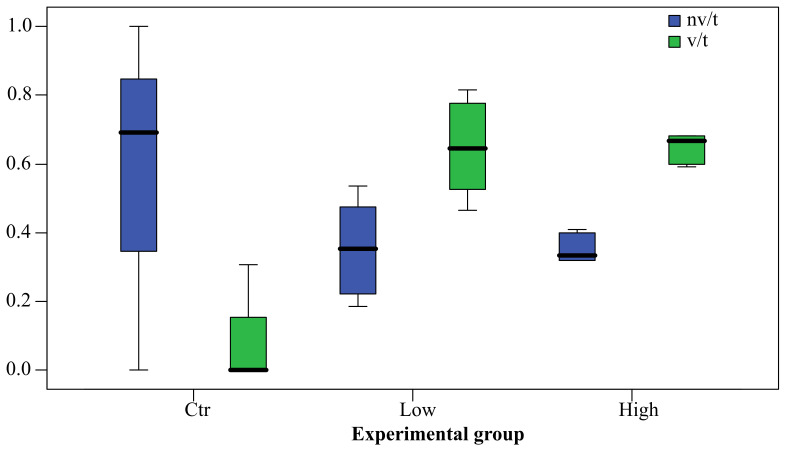
Box plot of the relative follicles number (in ordinate) according to morphology (nv/t = not vesiculated/total, v/t = vesiculated/total) and PFOA exposure group (Ctr, unexposed; Low concentration, 200 ng L^−1^ PFOA; High concentration, 2 mg L^−1^ PFOA). The bold line in the box represents the median and divides the box into a lower and upper quartile (25 percentile). The whiskers represent the upper and lower outer quartiles and, as a consequence, the upper and lower 50 percentile starting from the median.

Data on the absolute and relative follicle values are graphically summarized in Figure 2 and in Figure 3, respectively. PFOA exposure affected follicle occurrence and type. Both PFOA-exposed groups showed the total number of follicles (t) significantly higher compared to control group (Anova; *p* < 0.05). The same holds true for vesiculated follicles (v) (Kruskal-Wallis test; *p* < 0.05), whereas no significant difference was observed for not vesiculated follicles (nv). Furthermore, the relative follicle values were affected by treatment, but only for v/t, where both exposed groups displayed significant higher values compared to control (Kruskal-Wallis test; *p* < 0.05). With regard to follicle type within the same experimental group, there was a significant difference (t test; *p* < 0.05) between nv/t and v/t in the low concentration group, and between not vesiculated and vesiculated (t test; *p* < 0.01) and between nv/t and v/t (Wilcoxon test; *p* < 0.05) in the high concentration group. In summary, PFOA exposure was able to increase the occurrence of thyroid follicles in kidney, even in fish exposed to low concentration, in the tissues of which PFOA resulted below the LOD, and to affect their morphology, enhancing vesiculations. Moreover, in the high concentration group, where tissue PFOA concentrations were above LOD, the v/t value, and conversely the nv/t value, correlated positively (Spearman’s rho = 0.764; *p* < 0.05) and negatively (Spearman’s rho = −0.764; *p* < 0.05), respectively, with blood PFOA concentration (mean ± standard deviation; 64.87 ± 24.25), but not with renal PFOA concentration (mean ± standard deviation; 1.08 ± 0.54).

### 3.3. Follicle Ultrastructure

**Figure 4:**
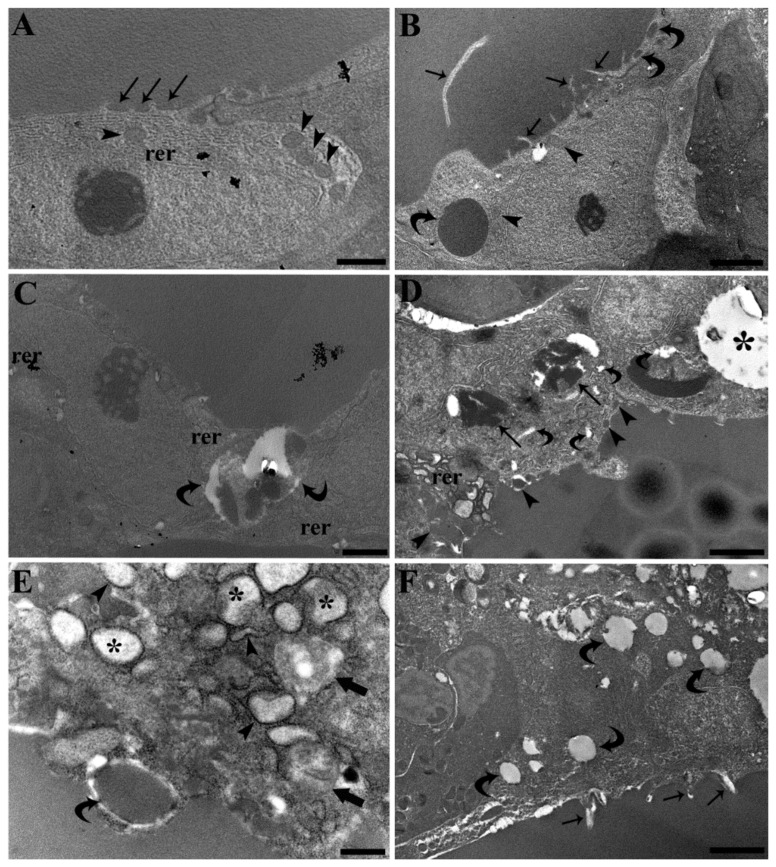
Transmission electron micrographs of ultrathin sections of common carp kidney. (**A**) A thyroid flattened pavement cell of an unexposed carp shows small colloid pinocytic caveolae (arrows), small mitochondria (arrowheads) and thin rough endoplasmic reticulum cisternae (rer). Scale bar = 1 μm. (**B**) Thyroid cuboidal cells in an unexposed carp display elongated cellular projections (arrows) and vesicles suggestive of colloid endocytosis (curved arrows). Mitochondria are also visible (arrowheads). Scale bar = 2 μm. (**C**) Phagolysosomes (curved arrows) and enlarged rough endoplasmic reticulum cisternae (rer) are appreciable in a cuboidal cell of an unexposed carp. Scale bar = 1 μm. (**D**) Thyroid cuboidal cells of a carp exposed to 200 ng L^−1^ PFOA display enhanced phagolysosomes formation (arrows), colloid endocytosis (arrowheads), enlargement and fragmentation of rough endoplasmic reticulum cisternae (rer), cytoplasm vacuolation (asterisk) and rarefaction (curved arrows). Scale bar = 2 μm. (**E**) The enlargement and fragmentation of rough endoplasmic reticulum cisternae are appreciable in a thyroid cuboidal cell of a carp exposed to 200 ng L^−1^ PFOA. Flocculent material is appreciable inside the cisternae (asterisks) and the persistence of ribosomes is a remarkable feature (arrowheads). Endocytized colloid (curved arrow) is also visible along with areas of cytoplasm rarefaction (arrows). Scale bar = 0.5 μm. (**F**) Thyroid cuboidal cells of a carp exposed to 2.0 mg L^−1^ PFOA display impressive cytoplasm vacuolations (curved arrows) and cellular projections (arrows). Scale bar = 2 μm.

Follicles of unexposed fish were lined by flattened pavement cells and/or cuboidal cells connected by junctional complexes. Cuboidal cells showed luminal cellular projections and phagocytic vacuoles, the content of which had the same ultrastructural appearance of follicle luminal colloids (Figure 4A,B). Phagolysosomes formation was also observed, along with a relative higher number of mitochondria and the extension of rough endoplasmic reticulum (consistent with functional cell hypertrophy) compared to pavement cells (Figure 4C).

Perfluorooctanoic acid exposure strongly affected follicle cell ultrastructure. Cellular projections, colloid phagocytosis and phagolysosomes formation increased. Rough endoplasmic reticulum enlargement and fragmentation were observed, along with the appearance of large vacuoles containing a clearer homogeneous content, distinguishable from colloid and suggestive of vacuolar degeneration. Moreover, areas of cytoplasm rarefaction were also observed. The two latter features were particularly evident in fish exposed to the highest PFOA concentration (Figure 4D–F). Accordingly, the enhanced cell vesiculation observed at light microscopy in PFOA-exposed fish has to be related to the following ultrastructural features: enhanced cellular projections; enhanced colloid phagocytosis; enhanced phagolysosomes formation; rough endoplasmic reticulum enlargement; and the occurrence of toxic cytoplasm vacuolation. The colloid vesiculation visible with light microscopy has to be ascribed the enhanced cytoplasm projections and the consequent local colloid rarefaction and/or possible resorption.

## 4. Discussion

The thyroid follicle structure and ultrastructure described in untreated fish agree with previous studies in cyprinids [16,17,25]. In teleostean fish, the thyroid is not a compact organ, being typically composed of follicles scattered within the subpharyngeal region, though the occurrence of “heterotopic” thyroid follicles has been reported. Among other anatomical sites, head and trunk kidneys are the most frequent “heterotopic” location, particularly in cyprinids [17]. With regard to common carp, though thyroid follicles are present in the subpharyngeal region, in the head and trunk kidneys the most hormonally active site is the latter, suggesting the reconsideration of the use of the term “heterotopic” for thyroid follicles outside the subpharyngeal region and to investigate further possible paracrine interplay among thyroid, hemopoietic, and excretory tissues [16,17]. Interestingly, in a previous study conducted on the same fish of the present research, PFOA exposure was shown to affect both the excretory and the hemopoietic renal tissues, with particular regard to rodlet cells, which are immune cells unique to teleost fish [18].

The thyroid disrupting effects of PFAS have been recently reviewed by Coperchini and collaborators [26], covering studies in vitro and on the animal model, along with data from occupational, general and reproductive medicine. Though in vitro and animal models studies support evidence of a thyroid disrupting effect, to date data are contrasting with regard to human beings [2,26]. Furthermore, it should be stressed that animal models rely mainly on endocrinological investigations [26], and there is a lack of previous research data on thyroid structural and ultrastructural effects as a consequence of chronic exposure at a relative low level of PFAS [11,25,27]. In particular, Chen et al. experimentally exposed zebrafish (*Danio rerio*) embryos at 8 h post fertilization to 250 µg l^−1^ of perfluorooctane sulfonic acid (PFOS) until 120 days post fertilization. Though no morphological changes were visible at light microscopy, the nuclear area of follicular epithelial cells was affected (reduced) by PFOS exposure. Moreover, vacuolation of mitochondria and the enlargement and degranulation of the rough endoplasmic reticulum were observed ultrastructurally [25]. The authors argued that ultrastructural changes may account for thyroid functional impairment and the related decline of thyroid hormone levels, though no further pathophysiological explanation was given [25]. Notably, the present study differs from that of Chen et al., by the following main aspects: PFOA was adopted instead of PFOS; adult fish were used instead of embryos; a fish species naturally occurring in in-land waters of the Eurasian continent and introduced throughout the world was used; an environmentally relevant concentration was tested; observed light microscopy changes were related to ultrastructural changes; and correlation was tested (at the highest used PFOA concentration) between structural changes and haematic PFOA concentration. As a consequence, the present study is better suited to deal with real environmental conditions in terms of biomarkers of exposure and effect, and in terms of the experimental model for thyroid disrupting effects in adult animals.

The observed PFOA-induced ultrastructural alterations should be regarded as signs of intra- and extracellular trafficking alteration, mainly involving thyroglobulin production and luminal excretion (exocrine phase), and resorption and cleavage (endocrine phase). Rough endoplasmic reticulum enlargement occurs commonly in many secretory cells, including thyroid cells [28,29]. Nevertheless, herein the enlargement is suggestive of misfolded protein retention within the rough endoplasmic reticulum leading to endoplasmic reticulum stress [28,30], though the latter occurrence should be investigated further by means of specific biomolecular markers. Actually, different endoplasmic reticulum storage diseases are known to affect the thyroid [31]. Interestingly, unfolded protein response and endoplasmic reticulum stress have already been proposed to be involved in the PFOA-induced hepatic pathophysiology in the same fish of this research [32], and have also been described in murine hepatic [33] and pancreatic cells [34] exposed to PFOA. Interestingly, RER enlargement was observed in zebrafish experimentally exposed to propylthiouracil, a drug used to inhibit new thyroid hormone production, by inhibiting the thyroid peroxidase enzyme [35]. Moreover, an endoplasmic reticulum storage disease characterized by molecular chaperones induction was reported in a human case of congenital hypothyroid goiter with deficient thyroglobulin [36]. Accordingly, RER enlargement should be regarded as the ultrastructural evidence of an impaired thyroid exocrine phase and thyroglobulin production. Contrary to this, enhanced cellular projections, colloid phagocytosis and phagolysosomes formation are in agreement with an increased thyroid stimulation and increased endocrine phase [29,37,38]. With regard to colloid vesiculations, they are traditionally considered to be the expression of hyperactivity and are frequently observed in otherwise normal thyroid in mammals, though evidences arose they could be related to apical plasma membrane and cytoplasm shedding into the follicle lumen, rather than representing enhanced colloid macropinocytosis [29,39]. Cytoplasm shedding is also a way the cell uses to get rid of damaged portions [40] and coexisting with signs of cytoplasm proteolysis, the areas of cytoplasm rarefaction, and of toxic vacuolation, suggests an incipient degenerative, toxic state, particularly in the high concentration group, rather than, or in addition to, follicle hyperactivity. The positive correlation reported between v/t value and blood PFOA concentration in the high concentration group is highly suggestive of the latter possibility and is deserving of further investigation. Alterations described herein in thyroid follicle cells were milder than those observed in the hepatocytes from the same fish of the present research, where mitochondria showed alterations like reduced or absent cristae, matrix lysis and vesiculation, swelling, and ballooning, along with the appearance of autophagosomes and myelin figures/myeloid bodies [41]. Milder alterations, compared to those in liver cells, were also observed in the nephron from the same fish of the present research, where signs of glomerular hyperfiltration, protein readsorbance by cells of the proximal tubular segment, and collecting duct mitochondria impairment were described [18]. Such a difference between liver and renal cell pathology was ascribed to the higher PFOA concentration measured in the liver compared to the kidney [18]. Though sharing the same anatomical tissue environment with renal cells, thyroid follicle cells did not show relevant mitochondria alterations, suggesting a somewhat different pathophysiology. Moreover, alterations were shown to be related to haematic but not renal PFOA concentration [18], stressing the existence of a microenvironment, in some way functionally separating thyroid follicles from renal cells, as suggested by the need of a strict epithelial-endothelial interactions in thyroid folliculogenesis [42]. Interestingly, PFOA was previously shown to affect the renal hemopoietic interstice, with particular regard to myeloid lineage cells and rodlet cells [18]. Furthermore, possible paracrine interaction between the thyroid and immune system was proposed by Geven and Klaren in carp kidney [17], suggesting that the aforementioned functional microenvironment may be shared by renal thyroid follicles and the renal hemopoietic interstice. Moreover, a possible thyroid/rodlet cells interaction should be specifically addressed in the future.

With regard to human health implications, care should be taken to avoid an oversimplified transposition of animal model data to human health [43]. Nevertheless, present results, with particular regard to the morphopathological evidences of a thyroid disrupting effect at a tissue concentration below the LOD, highlight the need to maintain the high threshold of attention, in particular in areas out of the known PFOA pollution hot spots, where human epidemiological data and clinical data may be lacking or not fully supporting evidence of a thyroid disrupting effect. According to the One Health approach, animals different from humans may be used as experimental models for human diseases (fish model) and as an indicator (sentinel) species for assessing the exposure of both humans and animals to environmental pollutants [13,14]. Accordingly, carp is a promising candidate as a sentinel fish for PFOA-induced thyroid disrupting effects and as a related fish model.

## 5. Conclusions

Perfluorooctanoic acid exposure was shown to affect the number, the structure and the ultrastructure of renal thyroid follicles, even at an environmental relevant concentration. A higher number of thyroid follicles and, in particular, the vesiculated ones, were observed in PFOA-exposed fish. Moreover, a positive correlation was reported between v/t value and blood PFOA concentration in the high concentration group, stressing the strict functional epithelial-endothelial (thyroid-blood) interactions proper of endocrine organs and confirming the thyroid disrupting effect of this pollutant in a fish model. Based on previous results about the role of carp kidney in monitoring the effect of PFOA exposure on both excretory and immune functions [18] and the latter observations, carp kidney candidates as a multipurpose biomarker organ in environmental pathology, in toxicologic pathology and in translational medicine.
